# The fate of redundant cues: Further analysis of the redundancy effect

**DOI:** 10.3758/s13420-014-0162-x

**Published:** 2014-12-24

**Authors:** Peter M. Jones, John M. Pearce

**Affiliations:** 1 School of Psychology, Cardiff University, Cardiff, UK; 2 School of Psychology, Plymouth University, Plymouth, PL4 8AA UK

**Keywords:** Associative learning, Blocking, Cue competition, Discrimination

## Abstract

Pearce, Dopson, Haselgrove, and Esber (*Journal of Experimental Psychology: Animal Behavior Processes*, 38, 167–179, 2012) conducted a series of experiments with rats and pigeons in which the conditioned responding elicited by two types of redundant cue was compared. One of these redundant cues was a blocked cue X from A+ AX+ training, whereas the other was cue Y from a simple discrimination BY+ CY–. Greater conditioned responding was elicited by X than by Y; we refer to this difference as the *redundancy effect*. To test an explanation of this effect in terms of comparator theory (Denniston, Savastano, & Miller, 2001), a single group of rats in Experiment 1 received training of the form A+ AX+ BY+ CY–, followed by an A– Y+ discrimination. Responding to the individual cues was tested both before and after the latter discrimination. In addition to a replication of the redundancy effect during the earlier test, we observed stronger responding to B than to X, both during the earlier test and, in contradiction of the theory, after the A– Y+ discrimination. In Experiment 2, a blocking group received A+ AX+, a continuous group received AX+ BX–, and a partial group received AX± BX± training. Subsequent tests with X again demonstrated the redundancy effect, but also revealed a stronger response in the partial than in the continuous group. This pattern of results is difficult to explain with error-correction theories that assume that stimuli compete for associative strength during conditioning. We suggest, instead, that the influence of a redundant cue is determined by its relationship with the event with which it is paired, and by the attention it is paid.

When two or more conditioned stimuli (CS) are presented simultaneously and paired with an unconditioned stimulus (US), some CSs may be more informative about the occurrence of the US than are others. A common finding is that, as learning progresses, the extent to which a CS provides information about the occurrence of the US will help to determine the extent to which it controls behavior. This effect is neatly demonstrated by the so-called *relative-validity effect*. In an experiment by Wagner, Logan, Haberlandt, and Price (1968), rats were presented with audio–visual compounds of AX and BX in one of two treatments. The continuous group received a simple discrimination in which AX was always followed by the US, and BX was never followed by the US (AX+ BX–). For the partial group, both compounds were followed by the delivery of the US, but on half of the trials only (AX±, BX±). In the former group, X was less informative about the occurrence of the US than either A or B; whereas A and B reliably predicted the presence and absence of the US, respectively, the US was equally likely to be present or absent following presentation of X. For the partial group, however, X was as informative as either of the other two cues. If the degree to which a cue comes to control behavior is determined by its relative informational value, then we should see greater behavioral control by X in the partial group than in the continuous group. This is precisely what Wagner et al. observed.

Another phenomenon that demonstrates that the informational value of a CS determines its influence on behavior is blocking. Kamin (1969) gave rats in a blocking group initial training in which a shock US was reliably signaled by an auditory CS, A (A+). In a second stage of training, a compound of A and a visual CS, X, was followed by the same US (AX+). We can think of X as being of no informational value, because the occurrence of the US was predicted by A, and X provided no extra information about whether or not the US would occur. Accordingly, Kamin found that X gained very little control over behavior. For comparison, a control group of rats was given AX+ training, but without prior A+ trials. In the latter case, we can see that A and X had equal statuses as predictors of the US, and behavioral control by X was accordingly greater than for those rats trained initially with A alone. The crucial factor here is that learning about X was determined by its informational value; for the blocking group, X was redundant, whereas for the control group, it was as informative as A. Indeed, Kamin found that if X in the blocking group provided some extra information about the US (achieved by increasing the intensity of the US on AX+ trials), then X gained substantial control over subsequent behavior, even after initial A+ training.

In relative-validity and blocking experiments, cues that are redundant in informational terms thus gain little control over behavior. The precise reasons for this outcome remain uncertain, however. A common assumption is that behavior is determined by learning about the relationships between a CS and a US, and that such learning about redundant cues is restricted in some way. According to Rescorla and Wagner (1972), for example, CSs compete for a limited amount of “associative strength,” and this competition restricts what is learned about redundant cues. In the case of the relative-validity experiment described above, AX+ BX– training will result in A gaining a substantial amount of positive associative strength, X gaining a small amount, and B gaining a small amount of negative associative strength that counteracts the small positive association between X and the US. In the case of blocking, initial A+ training allows A to gain substantial associative strength, restricting the quantity available on subsequent AX+ trials and leaving X with very little associative strength. Interestingly, this account makes a clear prediction about the relative associative strengths of the redundant cues in blocking and relative-validity tasks: An AX+ BX– discrimination will enable X to gain some associative strength, whereas A+ AX+ training will lead to X having no associative strength, provided that A+ training is complete. Thus, according to this analysis, blocking should be a more effective means than the simple discrimination for keeping to a minimum the associative strength of the redundant cue.

There are, however, both intuitive and empirical reasons to doubt this analysis. One feature of blocking, A+ AX+, is that X is reliably followed by the delivery of the US. The same is not true for AX+ BX– training. It might seem surprising, therefore, that X should be considered a poorer predictor of the US in the former case than in the latter. Additionally, recent attempts to test this prediction have produced the opposite result. Pearce, Dopson, Haselgrove, and Esber (2012) conducted experiments with both rats and pigeons, in which training of the form A+ AX+ BY+ CY– was given. This training contained both types of redundant cue already described. Learning about X thus should have been blocked by A, whereas the discrimination involving Y should have ensured that this cue was uninformative about the trial outcome. By including both types of redundant cue training in a single procedure, Pearce et al. were able to compare learning about them. The researchers found that the magnitude of the conditioned response (CR) was greater for the blocked cue X than for the irrelevant cue Y, in apparent contradiction of the model proposed by Rescorla and Wagner (1972). This result has been reproduced in humans (Uengoer, Lotz, & Pearce, 2013).

Pearce et al. (2012) considered several ways in which their results (henceforth referred to as the *redundancy effect*) can be accommodated by existing theories of learning, and the principal goal of this article is to present the first test in rats of two of these suggestions. The first possibility put forward by Pearce et al. was that cues in this task do not compete for associative strength during training. Instead, it is possible that each CS entered into association with the US in an independent manner. The proposition based on this is that learning is determined by an individual error term (e.g., Bush & Mosteller, 1951), as opposed to the summed error term that is a critical feature of the model proposed by Rescorla and Wagner (1972). If there is no competition between CSs for associative strength, then it is quite easy to see why X, with its history of being consistently paired with the US, should have elicited a stronger CR than Y, which was paired with the US only intermittently. The problem with this account is that it does not predict blocking. Without competition between the redundant and informative cues, there is no reason to suppose that learning about the former will be restricted. Fortunately, there is a theory that addresses this problem, by assuming that behavior is determined not only by learning, based on experience of the relationship between the CS and the US, but by an additional comparator process that acts at the time of testing. According to comparator theory (e.g., Denniston, Savastano, & Miller, 2001; Stout & Miller, 2007), the CR elicited by a stimulus will be determined by subtracting from its own associative strength some proportion of the associative strength of any other CSs with which it is associated. Informally, we can say that a cue will elicit a substantial CR if it has high associative strength relative to all of the stimuli with which it has previously been paired. In the case of blocking, A+ AX+, the high associative strength of A will, because of the comparator process, weaken considerably the CR elicited by X. This process may not, however, completely abolish the capacity for X to elicit a response, especially since this stimulus itself will have high associative strength. When blocking and a simple discrimination, BY+ CY–, are compared, because A and B are both reliably paired with the US, the subtractive effects of the comparator process can be expected to be similar for each of the redundant stimuli. However, because the associative strength of the intermittently reinforced Y will be less than that of the continuously reinforced X, the comparator process will result in a weaker response to Y than to X.

Not only can this account predict the redundancy effect, it also predicts the relative pattern of responding to the other cues presented in Pearce et al.’s (2012) experiments. For instance, consider B and X after training with A+ AX+ BY+ CY–. Like the Rescorla–Wagner (1972) model, comparator theory predicts that conditioned responding will be stronger for B than for X when the two are presented individually, despite both cues being consistently paired with the US. This is because B is associated with Y, a moderate predictor of the US, whereas X is associated with A, a good predictor of the US. This difference in the associative strengths of the comparison cues should produce the difference in the CRs to B and X. Although Pearce et al. did not test B on its own, Uengoer et al. (2013) conducted this test with human participants and observed precisely this pattern of results. Interestingly, it should be possible to test the comparator account by examining the consequences of further training with Y and A. If the difference in the CRs to B and X is a consequence of Y being a poorer predictor of the US than is A, we should be able to reverse this result by revaluing A and Y during subsequent A– Y+ training. The main purpose of Experiment 1 was to test this prediction.

Additionally, we note a feature of the training used by Pearce et al. (2012) that points to a theoretically uninteresting explanation for the redundancy effect. During A+ AX+ BY+ CY– training, each of the four trial types was presented with equal frequency. As a result, Y was presented twice as often as X. This difference in exposure to X and Y is potentially important, because recent examinations of attention during compound conditioning in rats have shown that the number of presentations of a cue can have a profound effect on the attention it is paid. For example, Jones and Haselgrove (2013) demonstrated that attention was lower for a blocking cue (such as A) than for a blocked cue (such as X), and that this difference could be eliminated by equating the numbers of occasions on which each of these cues was presented. If attention was highest for cues that had been presented on the fewest occasions during Pearce et al.’s experiments, we might anticipate that more learning would accrue to X than to Y. In Experiment 1, we sought to rule out this possibility by equating the numbers of presentations of X and Y.

## Experiment 1

A within-subjects design was used, which was based on Experiment 2 of Pearce et al. (2012) and is shown in Table 1. During the first stage of the experiment, rats received training with the A+ AX+ BY+ CY– discrimination, with the modification that the AX+ trials were presented twice as often as the other trial types. Animals thus received equal amounts of exposure to X and Y; if the difference in exposure between these cues was responsible for the redundancy effect demonstrated by Pearce et al., then we should fail to find this effect in the present study. This prediction was tested by subsequently presenting X and Y in isolation during Test 1. The second aim of this experiment was to test the extended comparator account of the redundancy effect, by examining how experience of an A– Y+ discrimination affects responding to B and X. Accordingly, we additionally presented B during Test 1, in order to make a baseline comparison with X. We expected the CR to be stronger to B than to X. In Stage 2, A– Y+ training was given, and this was followed by a further test of B and X. If the revaluation of A and Y was successful during Stage 2, the extended comparator account predicts that we should no longer see a stronger CR to B than to X during Test 2.

**Table 1:** The design of Experiment 1

Stage I	Tests 1 & 2	Stage II	Test 3
A+ AX+ BY+ CY–	B X Y	A– Y+	B X

During Stage 1, AX+ trials were presented with twice the frequency of the other trial types.

### Method

#### Subjects

The subjects were 24 experimentally naive, male Lister hooded rats supplied by Harlan Olac (Bicester, Oxon.). Before the start of the experiment, the rats were reduced gradually to 80 % of their free-feeding weights; they were subsequently maintained at these weights by being fed a controlled quantity of food after each daily experimental session. Rats were housed in pairs, in a lightproof room that was illuminated continuously for 14.5 h per day. They were tested at approximately the same time each day, with testing beginning 2 h after the onset of illumination.

#### Apparatus

The experiment was conducted in six identical conditioning chambers. The walls and ceiling of these boxes were made from transparent Perspex, and the floor consisted of a stainless steel grid. Each of the walls had a height of 23 cm above the grid floor, and a width of 30 cm. A circular hole of 3-cm diameter was cut into one of the walls, the center of which was 3 cm above the grid floor. This hole, which was equidistant from the two adjacent walls, provided access to a well into which sucrose solution (8 % sugar, 92 % water) could be delivered. This part of the apparatus is referred to as the *magazine*. Sucrose solution was delivered using a peristaltic pump situated beneath each experimental chamber; activation of this pump caused sucrose solution to be delivered, via a plastic tube, to the magazine. Experimental events, including the activation of the pump, were controlled via a PC with the Whisker control software (Campden Instruments Ltd, Loughborough, UK), which was programmed with VisualBasic 6.0. This computer also recorded the number of entries into the magazine, using information provided by infrared sensors that were attached to each chamber. Auditory stimuli were delivered simultaneously to all chambers via a 5-Ω speaker located on the ceiling of each chamber. Visual stimuli were presented to each rat on two adjacent flat-screen monitors with a width of 33 cm and a height of 27 cm, placed at an angle of 90° to each other. The point at which the two monitors met was 25 cm away from the nearest wall of the conditioning chamber, which was also the wall on which the magazine was located. The lower edge of each screen was at the same height as the floor of the chamber. Opaque screens were placed between the experimental chambers so that the subjects were unable to see each other.

Two auditory stimuli were used for the experiment: a 10-Hz, 76-dB clicker and a 2-kHz, 70-dB tone. Three visual stimuli were used: a blank white screen, a striped pattern consisting of alternating 3-cm black and white vertical bars, and a spotted pattern consisting of an 11 × 13 matrix of black circles with a diameter of 1.5 cm, on a white background. Each of the two black-and-white patterns filled the screen, and each visual stimulus was presented on each of the two screens in an experimental chamber simultaneously. These stimuli were counterbalanced such that for 12 of the subjects A, B, and C were the white, striped, and spotted screens, respectively; for six of these subjects, the tone served as X and the clicker served as Y, and for the remaining six, this arrangement was reversed. For the other 12 subjects, A, B, and C were the spotted, striped, and white screens, respectively. Again, the tone served as X and the clicker served as Y for half of these subjects, and this arrangement was reversed for the remainder.

#### Procedure

All of the subjects received two sessions of magazine training. During each 30-min session, sucrose was delivered to the magazine every 60 s. Each delivery consisted of 0.2 ml of solution, over a period of 3 s. Rats remained in the conditioning chambers for 30 min after the end of each of these sessions.

Stage I of the experiment consisted of 24 sessions, each containing 25 trials. Each subject received an A+, AX+, BY+, CY– discrimination, with ten presentations of AX during each session and five of each of the other trial types. These trials were presented in a random sequence, with the constraint that no more than two trials of the same type could occur consecutively. Each stimulus was presented for 10 s, with presentations of A, AX, and BY being followed immediately by a delivery of sucrose solution that was identical to those used during magazine training. The mean intertrial interval was 120 s (range = 80–160 s). Following this training, Tests 1 and 2 were administered. These tests each consisted of a single session, each containing 26 trials: 20 training trials of the type described above, and two nonreinforced test trials with each of X, Y, and B. The training trials were presented in random sequences within blocks of five trials, each containing two AX+ trials and one trial of each of the other types. Following the first two of these trial blocks, three test trials were administered. These were followed by a third block of training trials, a second block of test trials, and a final block of training trials. For half of the subjects, the first block of test trials consisted of the sequence X, B, Y, and the second block consisted of the sequence Y, B, X; for the remainder, this was reversed.

Stage II of the experiment consisted of an A–, Y+ discrimination, presented across four sessions. Each session contained 12 of each trial type in a random order, with the constraint that no more than two trials of each type could occur in succession. The other details of these sessions were the same as for Stage I. Test 3 consisted of a single session, containing 20 trials; the 11th, 12th, 17th, and 18th trials were nonreinforced presentations of B or X, and the remaining 16 trials were randomly sequenced presentations of A– or Y+. For half of the animals, the sequence of test trials was B, X, X, B, whereas for the remainder of the animals the sequence of test trials was X, B, B, X.

Throughout the experiment, the number of times that each rat entered the magazine during each 10-s trial, and during the 10 s before each trial, was recorded and taken as the measure of performance—referred to here as *response rate*. Although magazine training was successful for all subjects, two animals subsequently ceased responding during the experiment; the data from those animals are not included here.

### Results

**Fig. 1:**
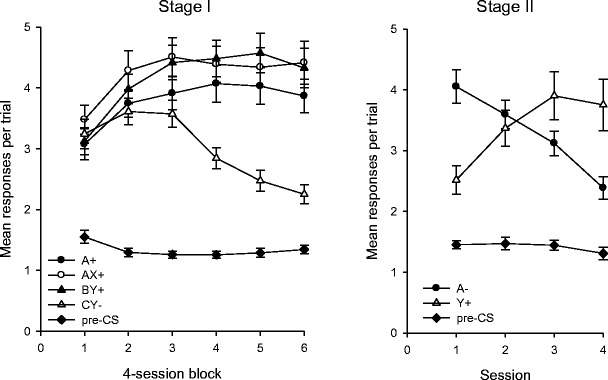
Mean rates of responding during Stages I and II of Experiment 1. Error bars show the standard errors of the means

Stage I conditioning proceeded smoothly, and the mean response rates for this stage are shown in the left-hand panel of Fig. 1. By the end of Stage I, the rate of responding was higher during the three reinforced trial types than during the nonreinforced presentations of CY. A one-way analysis of variance (ANOVA), conducted using each subject’s mean response rates during the final four sessions of Stage I, revealed a significant overall difference between the four trial types, *F*(3, 63) = 41.51, *MSE* = 0.54, *p* < .001. Subsequent *t* tests using a Bonferroni-corrected significance criterion revealed that responding during BY was equivalent to responding during AX, *t* < 1, but that all other comparisons produced a significant difference, smallest *t*(21) = 3.01, *p* = .007.

**Fig. 2:**
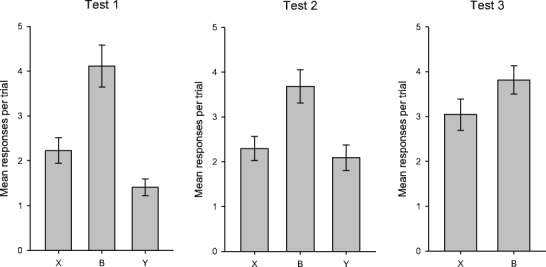
Mean rates of responding during Tests 1, 2, and 3 of Experiment 1. Error bars show the standard errors of the means

The results of Tests 1 and 2 are shown in the left-hand and center panels of Fig. 2. For Test 1, response rates were highest during B, and higher during X than during Y. A one-way ANOVA confirmed an overall difference among the three trial types, *F*(2, 42) = 35.30, *MSE* = 1.20, *p* < .001. Bonferroni-corrected *t* tests revealed that the rate of responding during X was lower than responding during B, *t*(21) = 5.42, *p* < .001, but that, crucially, responding during X was higher than responding during Y, *t*(21) = 3.50, *p* = .002. During Test 2, the response rates for the three trial types differed, *F*(2, 42) = 12.81, *MSE* = 1.23, *p* < .001. Although the rate of responding was lower during X than during B, *t*(21) = 3.80, *p* < .001, on this occasion there was no difference between the rates of responding during X and during Y, *t*(21) < 1. Comparing the mean response rates across Tests 1 and 2, the rate of responding was higher during X than during Y, *t*(21) = 2.30, *p* < .032. This difference between X and Y is a confirmation of the redundancy effect found by Pearce et al. (2012), except that in the present experiment the numbers of trials including X and Y during Stage I were equivalent.

The mean response rates during Stage II are shown in the right-hand panel of Fig. 1. This training was intended to extinguish conditioned responding to A and allow further conditioning to Y; a two-way ANOVA revealed no overall difference in the rates of responding during A and Y, *F*(1, 21) < 1, but a significant effect of session, *F*(3, 63) = 5.14, *MSE* = 0.36, *p* = .003, and a significant Stimulus × Session interaction, *F*(3, 63) = 32.93, *MSE* = 0.54, *p* < .001. Simple-effects analysis of this interaction showed that the rate of responding was higher during A than during Y for the first session, *F*(1, 84) = 20.11, *MSE* = 1.29, *p* < .001; that response rates during the two stimuli were equivalent for the second session, *F*(1, 84) < 1; and that the rate of responding was higher during Y than during A for each of the final two sessions, smaller *F*(1, 84) = 5.20, *MSE* = 1.29, *p* < .025. Additionally, we found significant effects of session for both stimuli, smaller *F*(3, 126) = 18.86, *MSE* = 0.45, *p* < .001. This analysis demonstrates that the revaluation procedure used during Stage II was effective, with the rates of responding during A and Y decreasing and increasing, respectively, such that the initial pattern of higher response rates during A than during Y was reversed. Following this revaluation, a final test was conducted; the results of Test 3 are shown in the right-hand panel of Fig. 2. Although the difference between the response rates during X and B was numerically smaller than it had been during the earlier test sessions, it was still statistically significant, *t*(21) = 2.13, *p* = .045. Additionally, we could detect no change in the response rates during X and B across tests. A two-way ANOVA was conducted to compare the average response rates in the first two tests with the response rates for Test 3, for both X and B. We found a significant effect of stimulus, *F*(1, 21) = 25.55, *MSE* = 1.25, *p* < .001, no effect of test, *F*(1, 21) < 1, and an interaction that approached but did not reach significance, *F*(1, 21) = 3.95, *MSE* = 1.04, *p* = .060. In light of the fact that the interaction was close to statistical significance, we also conducted separate analyses for each stimulus. However, we found no difference in responding during the final test and the earlier tests, for either X, *t*(21) = 1.94, *p* = .066, or B, *t*(21) < 1. The results of these comparisons were not dependent on the use of average response rates for Tests 1 and 2; equivalent results were obtained for *t* tests comparing the response rates during Test 3 with those from either Test 1 or Test 2.

The failure to observe either a reversal in the pattern of responding during X and B or any significant change in the response rates between tests is not consistent with predictions that can be derived from the formal model of comparator theory put forward by Stout and Miller (2007; see also Denniston et al., 2001). It might be argued, however, that the A– Y+ training in Stage 2 was not ideally suited for modifying the associative strengths of X and B in the manner predicted by comparator theory. First of all, Miller and Matute (1996) have argued that training in which BY is followed by food, prior to Y being paired with food, will not weaken responding to B as predicted by comparator theory, because the BY+ trials will leave B with high biological significance. Why the biological significance of B should influence the predictions of comparator theory is acknowledged by Stout and Miller (2007) to be beyond the scope of formal accounts of the theory. Second, there is evidence that if training of AX+ followed by A– is to enhance responding to X, then a large number of extinction trials with A are required (Blaisdell, Gunther, & Miller, 1999). Once again, it is not clear from comparator theory why a large number of extinction trials with A should be necessary in order for the response to X to be enhanced, but it is possible that the 56 nonreinforced trials with A were insufficient for this purpose. Thus, the results from the final test trials with B and X contradict the explanation offered by comparator theory for the redundancy effect, but whether this outcome is due to a shortcoming with the theory or to the manner in which the theory was tested remains to be determined.

Where does this leave us? The attraction of the extended comparator hypothesis was that it permitted us to suppose that learning might progress as a result of an individual error term rather than a summed error term. If we are to question this explanation, we must presumably also question the idea that learning is governed by an individual error term. We are, then, back to where we started—the summed error term incorporated into Rescorla and Wagner’s (1972) model predicts blocking, and can easily account for the failure of our revaluation procedure to reverse the difference in CRs to B and X (since Rescorla and Wagner’s, 1972, model predicts a much higher associative strength for B than for X, there is no reason to suppose that responding to these cues is dependent on the associative strengths of their associates). The initial problem remains, however—theories incorporating a summed error term are bound to predict that X will be blocked from acquiring associative strength by A, and that the CR to X will therefore be weaker than that to Y. Pearce et al. (2012) pointed out, however, that this assumption only holds if we assume that learning about A is at asymptote. In other words, if the acquisition of associative strength by A is incomplete, it is possible that some may be left for X. Whether this would allow X to acquire more associative strength than Y is another matter. Pearce et al. conducted a series of simulations of the Rescorla–Wagner model, using different values for the model’s parameters. In particular, they were interested in the relative values of *β*_E_ and *β*_I_, which are the learning rate parameters for trials with and without a US, respectively. In the case of a BY+ CY– discrimination, it follows that the acquisition of associative strength by Y will be most rapid when *β*_E_ > *β*_I_, and slowest when *β*_I_ > *β*_E_. The simulations conducted by Pearce at al. demonstrated that when *β*_I_ > *β*_E_, learning about Y may be sufficiently slow that X briefly gains more associative strength. This advantage is predicted be short-lived, because the associative strength of X will eventually be forced to zero as the associative strength of A approaches asymptote. Two conditions must therefore be met, if the redundancy effect is to be explained by the Rescorla–Wagner theory. The first condition is that learning about A should be incomplete, and the second is that learning should be more rapid on nonreinforced than on reinforced trials. As far as the first condition is concerned, Experiment 1 did not contain a definitive test of whether the associative strength of A had reached asymptote when testing with X took place. Nonetheless, it is worth noting that there were a total of 360 conditioning trials with A before testing began (120 with A+, 240 with AX+), and that responding was at the same high rate for the majority of these trials. Taken together, these observations imply that it is reasonable to assume that conditioning with A had reached asymptote when X was presented for testing and that, according to the Rescorla–Wagner theory, responding to X should therefore have been negligible.

Turning to the second condition, Rescorla (2002) has conducted a thorough examination of learning rates during appetitive conditioning in rats, and shown that changes in associative strength are greater for reinforced than for nonreinforced trials. Furthermore, the relative-validity effect can be explained by the Rescorla–Wagner model only if *β*_E_ > *β*_I_. Thus, the successful demonstrations of the relative-validity effect would seem to prevent this theory from explaining the redundancy effect. Of course, to date the redundancy effect in animals has been demonstrated in different experimental conditions from those prevailing in demonstrations of the relative-validity effect. Perhaps a crucial set of circumstances led to *β*_E_ being less *β*_I_ during tests of the redundancy effect, and to *β*_E_ being greater than *β*_I_ during tests of the relative-validity effect. A more compelling argument against the explanation derived from the Rescorla–Wagner theory for the redundancy effect might be made if the two effects were shown alongside one another, using conditions that were matched closely. This was the purpose of Experiment 2. It should be noted that Uengoer et al. (2013) conducted just such an experiment with human participants, and found that they were able to show both the redundancy effect and the relative validity effect in the same study. Nonetheless, it is important to reproduce this finding with rats.

## Experiment 2

The design of Experiment 2 is shown in Table 2. Three groups of rats were given a single training phase, followed by a test of the redundant cue X. For a blocking group, the training was of the form A+ AX+. The two remaining groups constituted a test of relative validity, with each receiving trials with AX and BX. For the continuous group, AX was consistently reinforced and BX was consistently nonreinforced. For the partial group, both compounds were followed by the US on half of the trials. Additionally, presentations of C were administered, which were nonreinforced for the blocking group but reinforced for the remaining two groups. The trials with C were included so that all three groups would receive the same overall rate of reinforcement during the experiment. The critical comparisons were the rates of responding during the test of X in the three groups. If responding was greater for the partial than for the continuous group, this would be a demonstration of the relative-validity effect and would indicate that, according to the Rescorla–Wagner (1972) model, the rate of learning for reinforced trials is greater than that for nonreinforced trials. If responding to X at test was also stronger for the blocking group than for the continuous group, this would be another demonstration of the redundancy effect. If both effects were seen, we would be unable to reconcile the overall pattern of results with the Rescorla–Wagner model.

**Table 2:** The design of Experiment 2

Group	Training	Test
Blocking	A+ AX+ C–	X
Continuous	AX+ BX– C+	X
Partial	AX± BX± C+	X

### Method

#### Subjects and apparatus

The subjects were 48 experimentally naive, male Lister hooded rats from the same stock and housed in the same manner as for Experiment 1. All rats were fed a restricted amount of food and maintained at 80 % of their free-feeding weights in the same way as in Experiment 1. These rats were divided randomly into three groups of equal size: the blocking group, the continuous group, and the partial group.

The apparatus used was the same as for Experiment 1, except for the assignments of the visual and auditory cues that served as experimental stimuli. For all rats, the clicker served as X and the white screen served as C. For half of the rats in each group, the spotted screen served as A and the striped screen served as B; for the remaining rats, this arrangement was reversed.

#### Procedure

Rats received two sessions of magazine training, according to the same procedure as in Experiment 1, followed by 20 sessions of discrimination training with 24 trials in each session. The rats in the blocking group received an A+, AX+, C– discrimination; the rats in the continuous group received an AX+, BX–, C+ discrimination; and the rats in the partial group received an AX±, BX±, C+ discrimination. For each group, each session consisted of eight trials of each type, presented in a sequence that was random, except for the constraint that no more than two trials of the same type could occur in succession. The other details of these sessions were the same as for Stage I of Experiment 1. Finally, all groups received a test session to assess the level of responding to X. This session contained four nonreinforced test trials with X, distributed amongst 20 training trials of the type described above (six trials with C, and seven of each of the other trial types). The test trials with X were administered as the 7th, 8th, 17th, and 18th trials of the session.

### Results

**Fig. 3:**
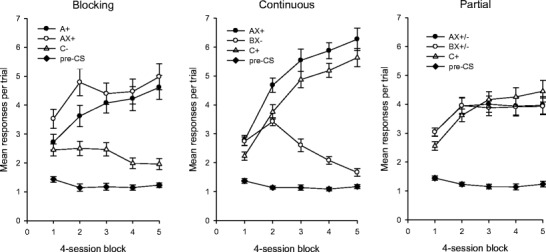
Mean rates of responding during the training stage of Experiment 2, for the blocking group (left panel), the continuous group (center panel), and the partial group (right panel). Error bars show the standard errors of the means

The mean response rates during the conditioning stage for each group are shown in Fig. 3. An analysis of the overall mean pre-CS rates for the three groups showed no between-group differences in the baseline rates of responding, *F* < 1. For the blocking group, a one-way ANOVA using the mean response rates during the final four sessions revealed an overall difference among the three trial types, *F*(2, 30) = 74.68, *MSE* = 0.58, *p* < .001. Bonferroni-corrected *t* tests showed that the rate of responding during A+ was lower than that during AX+, and that responding during C– was lower than that during each of the other two trial types, smallest *t*(15) = 2.92, *p* = .011. For the continuous group, we also found an overall difference among the three trial types, *F*(2, 30) = 135.69, *MSE* = 0.74, *p* < .001. Responding was lower during C+ than during AX+, and responding during BX– was lower than that for each of the other two trial types, smallest *t*(15) = 2.89, *p* = .011. For the partial group, there was an overall difference in response rates among the three trial types, *F*(2, 30) = 5.79, *MSE* = 0.24, *p* = .007. The rate of responding was lower during BX than during C, *t*(15) = 2.79, *p* = .014, but responding during AX was equivalent to that during both BX and C, larger *t*(15) = 2.39, *p* = .03.

**Fig. 4:**
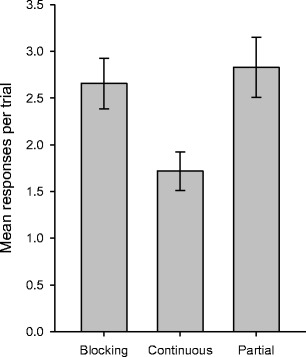
Mean rates of responding during the test stage of Experiment 2. Error bars show the standard errors of the means

The mean response rates during the final test with X are shown in Fig. 4. The initial impression, that the rate of responding was lower for the continuous group than for either the blocking or the partial group, was confirmed by the statistical analyses: A one-way ANOVA revealed an overall difference between the three groups, *F*(2, 45) = 4.87, *MSE* = 1.17, *p* = .012. Bonferroni-corrected *t* tests showed that the rate of responding was higher for the blocking than for the continuous group, *t*(30) = 2.75, *p* = .010, and lower for the continuous than for the partial group, *t*(30) = 2.90, *p* = .007. Both the redundancy effect and the relative-validity effect were therefore obtained, under closely matched conditions. This pattern of results cannot be predicted by the Rescorla–Wagner (1972) theory, irrespective of the values assigned to the parameters *β*_E_ and *β*_I_.

## General discussion

The two experiments reported here provide replications and extensions of the redundancy effect reported by Pearce et al. (2012), in which training with A+ AX+ BY+ CY– resulted in a stronger response to X than to Y. In Experiment 1, the redundancy effect was observed for the first time in animals when the numbers of presentations of X and Y were equated. In addition, the extended comparator theory (Denniston et al., 2001) was tested by comparing responding to B and to X, and examining the effects of revaluing A and Y. A stronger CR was recorded during B than during X, and this persisted following A– Y+ training. This result poses a serious obstacle if the theory is to account for our results. In Experiment 2, the redundancy effect was demonstrated alongside the relative-validity effect, which provides a pattern of results that is incompatible with the Rescorla and Wagner (1972) model, regardless of the values used for its parameters *β*_E_ and *β*_I_. These experiments therefore seem to rule out several established theories as explanations of our data.

A serious problem that confronts the Rescorla–Wagner (1972) model, when trying to explain the redundancy effect, is that it predicts that blocking will leave the blocked cue with no associative strength. It might seem, therefore, that any theory that does not make this prediction concerning blocking would be in a stronger position for explaining the redundancy effect. One such theory has been described by Pearce (1987, 1994), which predicts that even when conditioning with the blocking cue has reached asymptote, there is still scope for the blocked cue to excite a CR. However, simulations of this model that were described by Pearce et al. (2012) have revealed that it suffers from the same problems as those experienced by the Rescorla–Wagner theory when it must account for both the redundancy effect and the relative-validity effect. It would seem that we must therefore look beyond this theory for an adequate account of how redundant cues are treated.

Both Pearce et al. (2012) and Uengoer et al. (2013) have described experiments that test additional explanations of the redundancy effect. A quite obvious suggestion might be that the pattern of responding during X and Y is the result of associations formed between the CSs during initial training (see Rescorla & Durlach, 1981). As a result, the observed CR could be a product of not just the directly accrued associative strengths of X and Y, but also of those CSs with which they are associated. In order to accommodate our results, we would need to assume that associative activation of A by X had a more substantial effect on conditioned responding than did the activation of B and C by Y. Pearce et al. tested this account by revaluing A and C during A– C+ training and then conducting a further test of X and Y. During this test, the CR to X remained stronger than that elicited by Y, contrary to the prediction derived from considering within-compound associations.

As we discussed earlier, an intuitively plausible explanation for the redundancy effect is that the blocked cue, X, is consistently followed by a US, whereas the common cue from a simple discrimination, Y, is only intermittently paired with the US. This difference in reinforcement schedules leads naturally to the expectation that the US will be anticipated with more confidence when X rather than Y is presented. The results of a human predictive-learning experiment by Uengoer et al. (2013) indicated, however, that this explanation may be too simplistic. On each trial, participants were shown a picture of one or two foods (analogous to CSs) and told whether or not consumption of these foods resulted in a stomach ache (analogous to the US) for a hypothetical patient. The training phase of this experiment was based on the familiar form A+ AX+ BY+ CY–, but with additional D± DZ± trials, in which Z was intermittently paired with the outcome in the context of a blocking treatment. Following training, participants were asked to make judgments about the likelihood of a stomach ache following each of the different foods. Lower predictive ratings were given for Y than for X, a demonstration of the redundancy effect. Of particular interest, however, is the mean rating given for Z. If the low causal ratings given to Y were a result of its being followed by stomach ache only intermittently, then we should see causal ratings for Z that were no higher than those given for Y. The observed ratings for Z, however, were higher than those for Y (and equivalent to those for X). It seems, then, that the redundancy effect is not solely a consequence of the different effects of continuous and partial reinforcement. Instead, the influence of a redundant cue seems to be especially disrupted when it is present on both trials of a simple discrimination.

So far, we have considered that pairing a CS with a US is effective solely by changing the strength of an association between the internal representations of these events. It has been suggested on a number of occasions, however, that as learning about the relationships between a CS and US progresses, so the amount of attention paid to the former will change (e.g., Mackintosh, 1975a, 1975b; Pearce & Hall, 1980; Pearce & Mackintosh, 2010). Furthermore, these changes in attention are assumed to influence the ultimate strength of the CS–US association. Perhaps, then, the redundancy effect is at least partly a consequence of changes in attention, with more attention being focused on a blocked cue than on the redundant cue from the simple discrimination. This suggestion has some intuitive appeal, but specifying how exactly these changes in attention occur is not straightforward. Mackintosh’s (1975b) theory of attention might be particularly well suited to the present results, since changes in the associative strengths of CSs are, according to Mackintosh, governed by an individual error term. For this reason, learning about X may be more substantial than learning about Y. However, the rate of learning in Mackintosh’s (1975b) model is additionally determined by changes in attention, such that the attention paid to each CS is increased when it appears alongside poorer predictors of the US, and decreased when it appears alongside other CSs that are better predictors of the US. We would expect that the amount of attention paid to X would decrease substantially as a consequence of being trained alongside A, which is paired separately with the US. Changes in the attention paid to Y are more difficult to predict. This is because Y appears in varying circumstances, alongside cues that are more consistent predictors of either the presence of the US (in the case of BY+ trials) or its absence (CY– trials). Any overall change in attention to Y during this intermixed training is therefore rather difficult to specify, but it is at least possible that there could be a more substantial decrease in attention for Y than for X. This would enable Mackintosh’s (1975b) model to explain not just the present results, but the results of Uengoer et al. (2013) described above. However, Le Pelley (2004) pointed out that Mackintosh’s (1975b) model has difficulty explaining some other results, notably the development of conditioned inhibition. The source of this difficulty is the reliance of Mackintosh’s (1975b) model on an individual error term. To address this shortcoming, Le Pelley described a hybrid model that includes some features of Mackintosh’s (1975b) model, together with a summed error term (see also Pearce & Mackintosh, 2010). This hybrid approach permits explanation of a large number of attentional effects, but the presence of a summed error term makes Le Pelley’s model ill-suited for the present results; at the end of conditioning, a blocked cue should have less associative strength than the irrelevant cue from a simple discrimination. This problem is shared by other theories of attention that incorporate a summed error term (Esber & Haselgrove, 2011; Pearce & Hall, 1980).

Despite the difficulties in specifying how changes in attention take place, there is some evidence that this approach may be worth pursuing. Jones and Haselgrove (2013) compared the associabilities of A and X following A+ AX+ training, and found higher associability for X than for A. This is contrary to the predictions of the models of attention described above and suggests that, under at least some circumstances, attention is higher to blocked than to blocking cues. Likewise, Dopson, Esber, and Pearce (2010) compared the associabilities of A and X following AX+ BX– training, and found lower associability for X than for A. Unlike the blocked cue in Jones and Haselgrove’s experiments, the irrelevant cue in Dopson et al.’s experiments had lower associability than the relevant cue with which it was presented. Although it is difficult to draw any firm conclusions on the basis of a comparison of these two reports, it seems plausible that the associability of a blocked cue would be maintained better than that of the irrelevant cue from a simple discrimination. Additionally, evidence from studies of so-called *perceptual learning* (e.g., Blair & Hall, 2003; Hall, 2003; Mondragón & Hall, 2002) suggests that intermixed presentation of two similar compounds results in increased salience for the unique features of each compound, relative to the common features. In the present experiments, we can regard X as a unique feature in an intermixed A AX schedule, whereas Y is a common element in BY CY training. It is perhaps to be expected, then, that attention to X should be increased or maintained to a greater extent than attention to Y. In light of this evidence, and in the absence of a more compelling explanation for the redundancy effect, changes in the attention paid to redundant cues seem to merit further investigation.

In summary, the mechanisms underlying the redundancy effect remain unknown. The present experiments contribute to our understanding by eliminating several possibilities, without uncovering the true causes of this effect. Although we consider an explanation of this phenomenon to be a test of any purportedly complete theory of learning, it is also worth considering the possibility that a more complex approach is needed. Our efforts to uncover a simple set of rules that govern a broad range of learning situations should not be interpreted as reflecting a belief that learning itself must necessarily be simple. Where we are unable to accommodate a set of results within any given framework, we must entertain the idea that multiple interacting processes are at play.
